# What can Pakistan do to address maternal and child health over the next decade?

**DOI:** 10.1186/s12961-015-0036-5

**Published:** 2015-11-25

**Authors:** Zulfiqar A. Bhutta, Assad Hafeez

**Affiliations:** Center of Excellence in Women and Child Health, The Aga Khan University, Karachi, 74800 Pakistan; Health Services Academy, Islamabad, Pakistan

**Keywords:** Child health, Maternal health, Pakistan

## Abstract

Pakistan faces huge challenges in meeting its international obligations and agreed Millennium Development Goal targets for reducing maternal and child mortality. While there have been reductions in maternal and under-5 child mortality, overall rates are barely above secular trends and neonatal mortality has not reduced much. Progress in addressing basic determinants, such as poverty, undernutrition, safe water, and sound sanitary conditions as well as female education, is unsatisfactory and, not surprisingly, population growth hampers economic growth and development across the country. The devolution of health to the provinces has created challenges as well as opportunities for action. This paper presents a range of actions needed for change within the health and social sectors, including primary care, social determinants, strategies to reach the unreached, and accountability.

## Background

Pakistan is the sixth most populous country (185 million) in the world, with 64% of its people living in rural areas. Pakistan is currently experiencing a rapid population growth and, given the current rates, it will be the fifth most populous country globally by 2050 [1]. The overall literacy rate in the 15–45 year age group is 49% (males 56%, females 43%), with a life expectancy of 66.5 and 64.5 years for men and women, respectively [2]. Corresponding gains in the country’s health indicators, especially across maternal, newborn, and child health (MNCH) and nutrition, have fallen behind other low- and middle-income countries with comparable economies.

Pakistan’s maternal mortality ratio (MMR) has declined from 521 in 1990 to 332 (range, 250–433) in 2012, still far behind the proposed target of 130 by 2015 [3]. Complications of pregnancy and childbirth are the leading causes of death in women aged 15–45 years, accounting for 20% of the 8000 annual deaths among women of child-bearing age [3]. There are also huge disparities in maternal mortality: the MMR in rural parts of Pakistan is almost twice that in urban areas (319 vs. 175), and there are also wide variations between provinces, MMR being lowest in Punjab (227) and highest in Baluchistan (785) [4].

**Figure 1:**
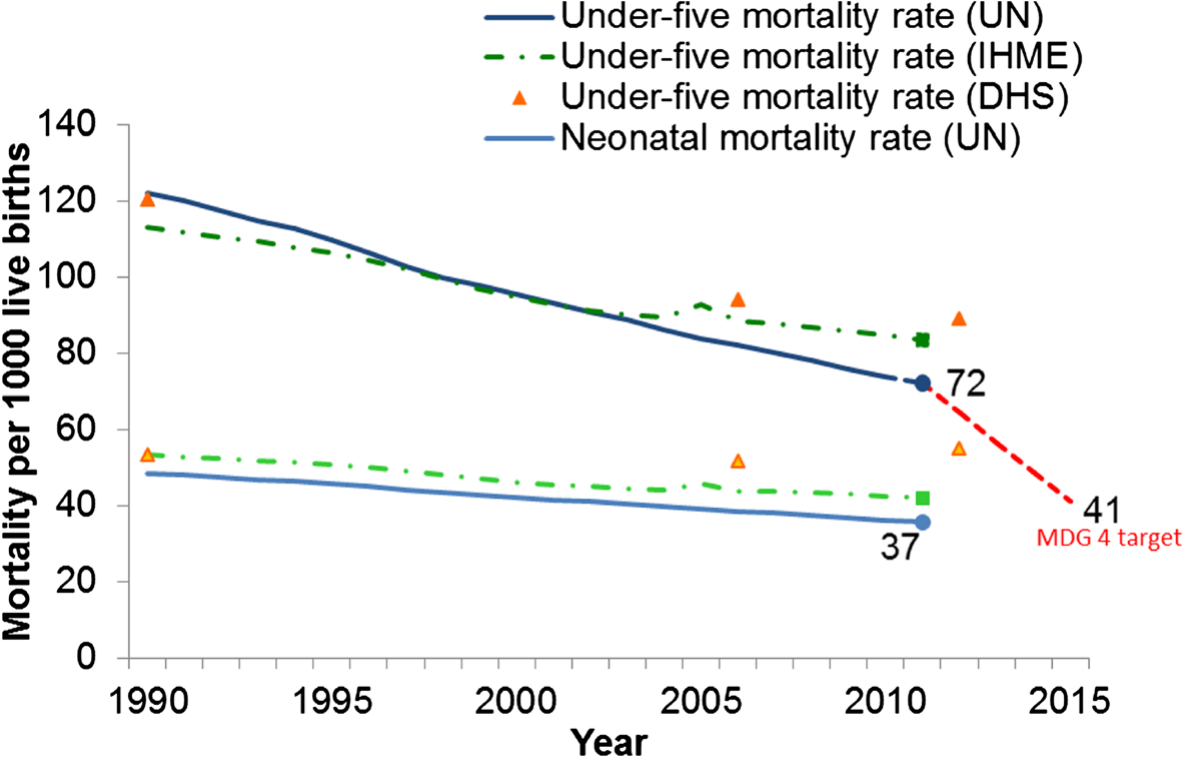
Under-5 mortality trends in Pakistan

Pakistan currently ranks 26th in the world for under-5 child mortality rates [5]. The under-5 mortality rate (per 1000 live births) has reduced from 141 in 1990 to 89 in 2012, but is much slower than the goal of reducing it to 46 by 2015 (Figure 1) [5, 6]. Around half of all under-5 deaths occur in the first month of life (202,000/year). After these neonatal deaths, diarrhoea, pneumonia, and malaria are the major causes of death of children under 5 worldwide [7], with clustering among low-birth weight or malnourished children. According to the Pakistan Demographic and Health Survey 2006–2007, the leading causes of death during the postnatal period are diarrhoea (27%) and pneumonia (26%), and are closely associated with overlapping risk factors such as poverty, under-nutrition, poor hygiene, and deprived home environments [4, 5].

The role of social determinants affecting MNCH in Pakistan cannot be underestimated. This is strongly influenced by socioeconomic characteristics, including place of residence, maternal education, and household wealth index. In Pakistan, there are wide disparities, and mortality rates in urban areas are consistently lower than in rural areas, although there are urban slums in mega-cities with equally high morbidity and mortality rates [2]. Nevertheless, despite wide perceptions of clustering of morbidity and mortality among urban slums, there is little data to this effect at the national level; among the poorest quintiles, under-5 mortality is 2.5 times higher (119) than in the richest (48) and there is also disparity amongst provinces: the under-5 mortality rate in Baluchistan is 111 and 70 in Khyber Pakhtunkhwa [2].

Under-5 mortality in children born to mothers with no education (112) is double that of children born to mothers with secondary education (57) and three-fold higher than children of mothers with more than a secondary education (36) [2]. Birth spacing and size/weight of an infant at birth are also important determinants of child mortality; an increase in the birth interval from 2–4 years or more results in better neonatal and child survival by 2.4 times in neonates to 2.9 times in children under 5 years [2]. Similarly, infants who are small at birth are twice as likely not to survive the neonatal period compared with infants of average or large size at birth.

In Pakistan, an estimated 21% of the population still lives below the poverty line, although these figures have been disputed as underestimates [8]. Other measures of poverty include food insecurity as well as rates of maternal and child undernutrition. Further, there are wide disparities between provinces and districts in rates of stunting and wasting among children as well as women of reproductive age who have a body mass index below 18.5 [8].

## What can be done?

Urgent action is needed to improve the state of MNCH through concerted, direct efforts, rather than expecting economic growth or poverty alleviation alone as the principle vehicles for change.

### Increased funding and allocation for MNCH

All provinces of Pakistan need to substantially increase funding for MNCH and nutrition, with the requisite multi-partisan support for sustainability. Pakistan’s current health spending is a mere 0.6% of the GDP. It is critical that investments in health and education and other social sectors increase substantially over the next few years [9]. Although health has been devolved to the provinces as a principal responsibility, inflation has eroded the corresponding increase in resources and support of primary health care programs. Much of the existing expenditure within the health sector is also limited to tertiary hospitals and, although primary care is supported through the lady health workers (LHW) program [10], there has been limited investment is strengthening district level health services, especially the Rural Health Centres and Basic Health Units. This must change from the current reliance on external assistance for these sectors to much more sustained and enhanced national and provincial funding for such efforts. Pakistan must target a significant increase in MNCH investments over the next 5 years.

### Enhancing the reach and quality of primary care services for MNCH in districts and urban slums

Primary care services in rural areas of Pakistan are critically dependent upon the LHW program and the relatively recent initiative to produce more community midwives [10]. The community midwifery program was developed as a consequence of the demand for provision of skilled care in poor rural populations with limited referral facilities. The recent, most formal evaluation of the LHW program and a number of informal reports post-devolution have identified several areas of weakness and opportunities for further enhancement of LHW skills and effective linkages with the People’s Primary Healthcare Initiative program [10], which manages the basic health units. Effective coverage has now become a serious issue given that there are managerial inefficiencies and between 30–50% of the population in several rural districts, especially the poorest and most remote areas, are without LHW cover. Similarly, although the community midwifery program was launched, it is unclear exactly how this has led to their retention and utility in these areas. There is a need for integrated services with linkages to vaccinators and other staff within the health system, as well as focusing the work of the LHWs on their core MNCH efforts as opposed to an increasing repertoire of services including non-communicable diseases and non-health sector activities [11].

### Improved quality of care in district facilities including rural health centres and district hospitals

This is a key barrier towards the promotion of care seeking in public sector facilities and is dependent upon a range of issues, including human resources, infrastructure, transportation, and communication. Establishing a linked referral chain for MNCH within the health system is dependent upon functioning facilities with provision of respectful care.

### Family planning

The vicious cycle of uncontrolled population growth and poor MNCH outcomes and nutrition in Pakistan needs urgent attention. The devolution of health and family planning to the provinces has created opportunities for integration; breaking down silos between hitherto parallel initiatives, such as the MNCH, expanded programme on immunization, and malaria and nutrition programs, is challenging and it is unclear if the federal ministry for health services and regulation will integrate national oversight of this. It is naive to imagine that gains in MNCH and nutrition can be achieved by intervening within the health sector alone.

### Investing in addressing social determinants of health

There is also the urgent need to tackle social determinants affecting MNCH in Pakistan, which relate to fundamental issues of the status of women, their education and empowerment, the built environment, and water, sanitation, and hygiene [12]. Given the critical role of maternal education in improving child survival and maternal health, there is an urgent need for these investments. Integrating health and development messages in linking MNCH to other sectors, such as education, prevention of child marriage, and gender empowerment, are a key task for the federal ministry in tandem with the provinces. Further, enhanced efforts to address maternal and child undernutrition with a focus on adolescent girls are needed given the critical need to address food insecurity and undernutrition. Notwithstanding the importance of nutrition-sensitive interventions, the role of the health sector in enabling the implementation of nutrition-specific interventions is critical; this is especially true for integrating maternal nutrition and breastfeeding support strategies, ensuring that major causes of micronutrient deficiencies are addressed, and that nutrition prevention and promotion is integrated within the primary care programs.

### Measurement and accountability at the district level

One of the key limitations for action is the lack of accurate and timely information. Pakistan has long depended upon expensive and time-consuming cross-sectional surveys for assessment of progress and to-date key information on important issues of direct causes of mortality and morbidity are not available at the provincial level. There is a need for strengthening of district information systems, such as the District Health Information Software, and the creation of sentinel information systems for important areas related to MNCH by age group and gender. There is a corresponding need for a national multi-stakeholder body for oversight of MNCH and nutrition in addition to the important role of the Federal Ministry for Health Services and Regulation.

## Conclusions

Finally, with the emerging consensus on sustainable development goals and universal health coverage [12], Pakistan’s quest for improving its MNCH indicators is only likely to succeed if the federal and provincial leaderships are cognizant of the importance of these investments for national development. MNCH and nutrition need to be national priorities clearly understood by the ministries of finance, economic affairs division, and the planning commission.

Despite widespread perceptions of a failed state and a crisis of governance, we believe that the situation in Pakistan is ripe for change. While successive leaders may have failed the common man, civic society in Pakistan is vibrant and democracy is slowly but steadily taking roots. The 18th Amendment [5] and devolution of health to provinces also offers a unique opportunity for focusing attention on the health and nutrition of women and children where it matters, at the district level. The key would be the implementation of a primary care strategy for women and children as a fundamental strategy for investing in the nation’s future.

## Abbreviations

LHW: Lady health workers
MMR: Maternal mortality ratio
MNCH: Maternal, newborn, and child health
